# DEVELOPMENT AND VALIDATION OF THE ANTIPSYCHOTIC MEDICATION RISK SCORE FOR NURSING HOME RESIDENTS

**DOI:** 10.1093/geroni/igz038.3310

**Published:** 2019-11-08

**Authors:** Lorraine J Phillips, Greg Petroski, Nancy Birtley

**Affiliations:** 1 University of Delaware, Newark, Delaware, United States; 2 University of Missouri, Columbia, Missouri, United States

## Abstract

Prevalence rates of antipsychotic medication (APM) use in U.S. long-stay nursing home (NH) residents, excluding those with approved diagnoses, range from 7.2% to 20.7%; Missouri’s rate is 18.6%. This study developed an APM risk score for NH residents using variables from the Minimum Data Set 3.0 (MDS 3.0) assessment. Data from the most recent Missouri MDS 3.0 assessment, excluding admission and discharge, for each long-stay NH resident from November 2017–December 2018 were used to create development (n= 30,893) and validation (n= 7,651) data sets. Potential predictors of APM use were entered in a logistic regression model with variable selection via the least absolute shrinkage and selection operator (LASSO). In a final step, only variables with odds ratios > 1.2 were retained. A weighted score was created by assigning points relative to the maximum coefficient [10*βi /max (β)] and rounded to integer values. APM rates were 17.29% and 17.70% in the development and validation data, respectively. The final model included 14 demographic and clinical indicators; assigned points (1-10) summed for total score (0-50). Areas under receiver operator characteristic curves were 0.801 and 0.798 for the development and validation models, respectively. Youden's index cut-point = 8, with sensitivity of .70 and specificity of .75. Our findings demonstrate it is possible to predict with good accuracy a NH resident’s risk of APM use. Identifying residents at increased risk of receiving an APM, perhaps inappropriately, could position NH staff to proactively design and deliver nonpharmacological interventions individualized to each resident’s needs and preferences.

